# MicroRNAs: Fine Tuners of Monocyte Heterogeneity

**DOI:** 10.3389/fimmu.2019.02145

**Published:** 2019-09-23

**Authors:** Isabelle Duroux-Richard, Maxime Robin, Cindy Peillex, Florence Apparailly

**Affiliations:** ^1^IRMB, INSERM, University of Montpellier, Montpellier, France; ^2^Clinical Department for Osteoarticular Diseases, University Hospital of Montpellier, Montpellier, France

**Keywords:** microRNA, monocytes, Ly6C^high^, Ly6C^low^, CD14^+^, CD16^+^

## Abstract

Small non-coding microRNAs (miRNAs) have been found to play critical roles in many biological processes by controlling gene expression at the post-transcriptional level. They appear to fine-tune the immune response by targeting key regulatory molecules, and their abnormal expression is associated with immune-mediated inflammatory disorders. Monocytes actively contribute to tissue homeostasis by triggering acute inflammatory reactions as well as the resolution of inflammation and tissue regeneration, in case of injury or pathogen invasion. Their contribution to tissue homeostasis can have many aspects because they are able to differentiate into different cell types including macrophages, dendritic cells, and osteoclasts, which fulfill functions as different as bone remodeling and immune response. Monocytes consist of different subsets with subset-specific expression of miRNAs linked to distinct biological processes dedicated to specific roles. Therefore, understanding the role of miRNAs in the context of monocyte heterogeneity may provide clues as to which subset gives rise to which cell type in tissues. In addition, because monocytes are involved in the pathogenesis of chronic inflammation, associated with loss of tissue homeostasis and function, identifying subset-specific miRNAs might help in developing therapeutic strategies that target one subset while sparing the others. Here, we give an overview of the state-of-the-art research regarding miRNAs that are differentially expressed between monocyte subsets and how they influence monocyte functional heterogeneity in health and disease, with descriptions of specific miRNAs. We also revisit the existing miRNome data to propose a canonical signature for each subset.

## Introduction

MicroRNAs (miRNAs) are a class of short non-coding RNAs (18–22 nt), conserved from worms to mammals that play a regulatory role in gene expression at the posttranscriptional level (1). Since their discovery, many studies have shown that they are involved in biological processes. Quantitative and qualitative assessments of miRNA expression in various disease conditions have revealed considerable changes in their expression profiles.

The biogenesis of miRNAs occurs in the nucleus. MiRNA-encoding genes are transcribed to a primary miRNA and processed by Drosha, a class 2 RNase III enzyme, into a precursor miRNA (pre-miRNA), which is exported to the cytoplasm by exportin-5. In the cytoplasm, mature forms of miRNAs are produced after several steps involving Dicer, a RNase III type protein, and RISC, a RNA-induced silencing complex (2). MiRNA genes can be located in the context of non-coding transcription units or in the introns of protein-coding genes (3, 4). Almost half of miRNA genes are clustered and can be independently or simultaneously transcribed into single polycistronic transcripts (5, 6).

Currently, more than 2,800 and 2,100 miRNAs have been identified in human and mouse, respectively (miRBase vs22). Both *in vitro* and bioinformatic analyses have determined that more than 500 genes could be targeted by a single miRNA (7, 8). MiRNAs bind mRNA targets by their “seed” sequence interacting with the 3′untranslated region (UTR), and more rarely with the coding region (CDS) or 5′UTR, of the targeted mRNA (9). According to the degree of complementarity, miRNAs lead to mRNA cleavage and degradation or to the inhibition of translation, thus interfering with the downstream protein output (10). MiRNA family members can be highly conserved among vertebrates, in particular in the seed region, which corresponds to nucleotides 2 to 7/8 and is the main determinant of target specificity (11). Thus, miRNAs with similar seed sequence can target similar sets of genes and similar biological pathways.

Extensive work has been performed to identify miRNA-specific signatures in immune cells and to understand how a specific miRNA gene controls the development and function of a specific immune cell population. However, few studies have addressed the role of miRNAs in terms of subset heterogeneity of one specific immune cell type. Here we review reports of miRNAs in monocyte subsets and performed an *in silico* analysis that also includes new data to revisit the current knowledge of monocyte subset functions.

## miRNA-Based Signatures Specific to Monocyte Subsets

Monocytes are composed of two main subsets in both mouse and human (12) that are committed to different functions (13–15): in mice, the “classical” inflammatory Ly6C^high^ and the “non-classical” patrolling Ly6C^low^ monocyte subsets. Their human counterparts are CD14^+^CD16^−^ and CD14^dim^CD16^++^, respectively (12, 13). Ly6C^high^ monocytes secrete inflammatory mediators in response to bacteria and can differentiate into macrophages, inflammatory dendritic cells (DCs), and osteoclasts (OCs) (16–18). Ly6C^low^ monocytes survey endothelial cells and surrounding tissues to detect damage and viral threat and are involved in tissue repair (13). Although mouse and human studies have underscored the relevance of studying monocyte subsets in disease by showing differential accumulation of both subsets, factors that regulate monocyte subset fate and functional heterogeneity under pathophysiological conditions remain poorly explored.

MiRNAs play pivotal roles in regulating monocyte development and functions, including differentiation, tissue recruitment, activation, initiation, and resolution of inflammation (19); however, very little is known about their involvement when considering the heterogeneity of monocytes. An attempt to close this gap in knowledge has been addressed by miRNome analyses of monocyte subsets. Nevertheless, few miRNome analyses of monocyte subsets have been performed with human or mouse samples. To our knowledge and from free-access databases, we identified only three studies (20–22); two focused on miRNAs differently expressed between classical and non-classical monocytes, in humans and mice, without considering “intermediate” monocytes (20, 21). After showing differences in DNA methylation in the three human monocyte subsets—classical, non-classical, and intermediate (23)—Zawada et al. studied miRNA profiling for human “intermediate” monocytes (22).

Thus, in the current review, we combined all existing data with our own unpublished miRNome data for both classical and non-classical monocyte subsets isolated from human and mouse blood to provide novel insights into monocyte subset-specific miRNA signatures.

### Analysis of the miRNome of Classical and Non-classical Monocyte Subsets

**Figure 1:**
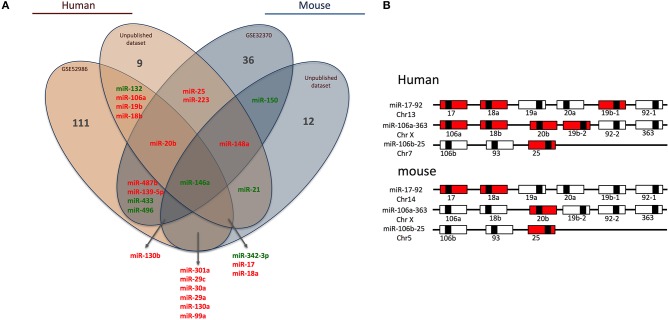
Human and mouse miRNome profiles identifying monocyte subset-specific miRNAs. **(A)** Four datasets were analyzed and clustered to obtain a Venn diagram showing miRNAs differentially regulated between classical and non-classical monocytes and common to human and mouse. Red and green represent miRNAs up- and down-regulated, respectively, in classical vs. non-classical monocytes. **(B)** Schematic representation of members of the miR-17/92 family of miRNA gene clusters in human and mouse. MiRNAs upregulated in classical monocytes vs. non-classical monocytes are in red.

Briefly, we collected miRNome datasets (GSE52986 and GSE32370) from the GEO bank (http://www.ncbi.nlm.nih.gov/geo/). For each GEO dataset, we compared miRNA expression profiles for classical and non-classical monocyte subsets (i.e., CD14^++^ CD16^−^ and CD14^+^ CD16^++^ for human blood samples, Ly6C^high^ and Ly6C^low^ for mouse blood samples) to obtain a list of miRNAs differentially expressed between the subsets in both species. The technical platforms used in these two studies were Illumina Human v2 and Mouse v1 MicroRNA expression beadchips, respectively. Also, we performed large-scale miRNA screening using a TaqMan low-density array to identify miRNAs differentially expressed between classical and non-classical monocyte subsets in human and mouse. With false discovery rate-adjusted *P* ≤ 0.05, we found 25 miRNAs differentially expressed between classical and non-classical monocytes. We then used a Venn diagram to visualize common miRNAs between all four datasets (http://www.irp.nia.nih.gov/bioinformatics/vennplex.html). Only miR-146a was commonly downregulated in classical monocytes, for all available human and mouse datasets, independent of the technological platform used (Figure 1A). At the intersection of human datasets, we identified nine miRNAs (miR-132, miR-106a, miR-19b, miR-18b, miR-20b, miR-146a, miR-342-3p, miR17, and miR18a): miR-132, miR146a, and miR-342-3p showed lower expression in classical than non-classical monocytes. At the intersection of mouse datasets, we identified 4 miRNAs (miR-146a, miR-130b, miR-150, and miR-148a); miR-148a and miR-130b were upregulated in Ly6C^high^ versus Ly6C^low^ monocytes. Only a very small number of miRNAs was specific to mouse (miR-150) or human (miR-18b, miR-19b, miR-106a, and miR-132) subsets.

### Genomic Organization of miRNAs Specific to Monocyte Subsets

Among the 25 miRNAs identified, 16 showed sequence homology between human and mouse (Table 1) and almost 70% were organized in clusters in both species; examples are miR-17/92, miR-106a/363 and miR-106b/25 (Figure 1B). Only two miRNAs were not organized in clusters in either species: miR-342/151b and miR-150/5121 in human and mouse, respectively.

**Table 1:** List of miRNAs common in human and mouse miRNome datasets.

**Name**	**Species**	**Cluster (miRNA)**	**Chromosome position**	**miRNA mature seq**	miRNA* mature seq
miR-106a	*hsa*	Yes (miR-18b, miR-20b, miR-19b-2, miR-92a-2, miR-363)	chrX: 134170198-134170278	AAAAGUGCUUACAGUGCAGGUAG	CUGCAAUGUAAGCACUUCUUAC
	*mmu*	Yes (miR-18b, miR-20b, miR-19b-2, miR-92a-2, miR-363)	chrX: 52742503-52742567	CAAAGUGCUAACAGUGCAGGUAG	ACUGCAGUGCCAGCACUUCUUAC
miR-130a	*hsa*	No	chr17: 59151136-59151221	GCUCUGACUUUAUUGCACUACUCAGUGCAAUAGUAUUGUCAAAGC	
	*mmu*	No	chr11: 87113004-87113089	CAGUGCAAUAGUAUUGUCAAAGC	GCUCUGACUUUAUUGCACUACU
miR-130b	*hsa*	yes (miR-301b)	chr22: 21653304-21653385	CAGUGCAAUGAUGAAAGGGCAU	ACUCUUUCCCUGUUGCACUAC
	*mmu*	yes (miR-301b)	chr16: 17124061-17124142	CAGUGCAAUGAUGAAAGGGCAU	ACUCUUUCCCUGUUGCACUACU
miR-132	*hsa*	Yes (miR-212)	chr17: 2050271-2050380	UAACAGUCUACAGCCAUGGUCG	ACCGUGGCUUUCGAUUGUUACU
	*mmu*	Yes (miR-212)	chr11: 75173388-75173478	UAACAGUCUACAGCCAUGGUCG	AACCGUGGCUUUCGAUUGUUAC
miR-139-5p	*hsa*	No	chr11: 72615063-72615130	UCUACAGUGCACGUGUCUCCAGU	
	*mmu*	No	chr7: 101475376-101475443	UCUACAGUGCACGUGUCUCCAG	
miR-146a	*hsa*	No	chr5: 160485352-160485450	UGAGAACUGAAUUCCAUGGGUU	CCUCUGAAAUUCAGUUCUUCAG
	*mmu*	No	chr11: 43374397-43374461	UGAGAACUGAAUUCCAUGGGUU	CCUGUGAAAUUCAGUUCUUCAG
miR-148a	*hsa*	No	chr7: 25949919-25949986	UCAGUGCACUACAGAACUUUGU	AAAGUUCUGAGACACUCCGACU
	*mmu*	No	chr6: 51269812-51269910	UCAGUGCACUACAGAACUUUGU	AAAGUUCUGAGACACUCCGACU
miR-150	*hsa*	No	chr19: 49500785-49500868	UCUCCCAACCCUUGUACCAGUG	CUGGUACAGGCCUGGGGGACAG
	*mmu*	yes (miR-5121)	chr7: 45121757-45121821	UCUCCCAACCCUUGUACCAGUG	CUGGUACAGGCCUGGGGGAUAG
miR-17	*hsa*	Yes (miR-18a, miR-19a, miR-20a, miR-19b-1, miR-92a-1)	chr13: 91350605-91350688	CAAAGUGCUUACAGUGCAGGUAG	ACUGCAGUGAAGGCACUUGUAG
	*mmu*	Yes (miR-18a, miR-19a, miR-20a, miR-19b-1, miR-92a-1)	chr14: 115043671-115043754	CAAAGUGCUUACAGUGCAGGUAG	ACUGCAGUGAGGGCACUUGUAG
miR-18a	*hsa*	Yes (miR-17, miR-19a, miR-20a, miR-19b-1, miR-92a-1)	chr13: 91350751-91350821	UAAGGUGCAUCUAGUGCAGAUAG	ACUGCCCUAAGUGCUCCUUCUGG
	*mmu*	Yes (miR-17, miR-19a, miR-20a, miR-19b-1, miR-92a-1)	chr14: 115043851-115043946	UAAGGUGCAUCUAGUGCAGAUAG	ACUGCCCUAAGUGCUCCUUCUG
miR-18b	*hsa*	Yes (miR-106a, miR-20b, miR-19b-2, miR-92a-2, miR-363)	chrX: 134170041-134170111	UAAGGUGCAUCUAGUGCAGUUAG	UGCCCUAAAUGCCCCUUCUGGC
	*mmu*	Yes (miR-106a, miR-20b, miR-19b-2, miR-92a-2, miR-363)	chrX: 52742331-52742413	UAAGGUGCAUCUAGUGCUGUUAG	UACUGCCCUAAAUGCCCCUUCU
miR-19b-1	*hsa*	Yes (miR-17, miR-19a, miR-18a, miR-20a, miR-92a-1)	chr13: 91351192-91351278	UGUGCAAAUCCAUGCAAAACUGA	AGUUUUGCAGGUUUGCAUCCAGC
	*mmu*	Yes (miR-17, miR-19a, miR-18a, miR-20a, miR-92a-1)	chr14: 115044305-115044391	UGUGCAAAUCCAUGCAAAACUGA	AGUUUUGCAGGUUUGCAUCCAGC
miR-19b-2	*hsa*	Yes (miR-106a, miR-18b, miR-20b, miR-92a-2, miR-363)	chrX: 134169671-134169766	UGUGCAAAUCCAUGCAAAACUGA	AGUUUUGCAGGUUUGCAUUUCA
	*mmu*	Yes (miR-106a, miR-18b, miR-20b, miR-92a-2, miR-363)	chrX: 52741983-52742066	UGUGCAAAUCCAUGCAAAACUGA	AGUUUUGCAGAUUUGCAGUUCAGC
miR-20b	*hsa*	Yes (miR-106a, miR-18b, miR-19b-2, miR-92a-2, miR-363)	chrX: 134169809-134169877	CAAAGUGCUCAUAGUGCAGGUAG	ACUGUAGUAUGGGCACUUCCAG
	*mmu*	Yes (miR-106a, miR-18b, miR-19b-2, miR-92a-2, miR-363)	chrX: 52742113-52742192	CAAAGUGCUCAUAGUGCAGGUAG	ACUGCAGUGUGAGCACUUCUAG
miR-21	*hsa*	No	chr17: 59841266-59841337	UAGCUUAUCAGACUGAUGUUGA	CAACACCAGUCGAUGGGCUGU
	*mmu*	No	chr11: 86584067-86584158	UAGCUUAUCAGACUGAUGUUGA	CAACAGCAGUCGAUGGGCUGUC
miR-223	*hsa*	No	chrX: 66018870-66018979	UGUCAGUUUGUCAAAUACCCCA	CGUGUAUUUGACAAGCUGAGUU
	*mmu*	No	chrX: 96242817-96242926	UGUCAGUUUGUCAAAUACCCCA	CGUGUAUUUGACAAGCUGAGUUG
miR-25	*hsa*	Yes (miR-106b, miR-93)	chr7: 100093560-100093643	CAUUGCACUUGUCUCGGUCUGA	AGGCGGAGACUUGGGCAAUUG
	*mmu*	Yes (miR-106b, miR-93)	chr5: 138165321-138165404	CAUUGCACUUGUCUCGGUCUGA	AGGCGGAGACUUGGGCAAUUGC
miR-29a	*hsa*	Yes (miR-29b-1)	chr7: 130876747-130876810	UAGCACCAUCUGAAAUCGGUUA	ACUGAUUUCUUUUGGUGUUCAG
	*mmu*	Yes (miR-29b-1)	chr6: 31062660-3106274	UAGCACCAUCUGAAAUCGGUUA	ACUGAUUUCUUUUGGUGUUCAG
miR-29c	*hsa*	Yes (miR-29b-2)	chr1: 207801852-207801939	UAGCACCAUUUGAAAUCGGUUA	UGACCGAUUUCUCCUGGUGUUC
	*mmu*	Yes (miR-29b-2)	chr1: 195037547-195037634	UAGCACCAUUUGAAAUCGGUUA	UGACCGAUUUCUCCUGGUGUUC
miR-30a	*hsa*	No	chr6: 71403551-71403621	UGUAAACAUCCUCGACUGGAAG	CUUUCAGUCGGAUGUUUGCAGC
	*mmu*	No	chr1: 23272269-23272339	UGUAAACAUCCUCGACUGGAAG	CUUUCAGUCGGAUGUUUGCAGC
miR-342-3p	*hsa*	Yes (miR-151b)	chr14: 100109655-100109753	UCUCACACAGAAAUCGCACCCGU	
	*mmu*	No	chr12: 108658620-108658718	UCUCACACAGAAAUCGCACCCGU	
miR-433	*hsa*	Yes (miR-337, miR-665, miR-431, miR-127, miR-432, miR-136)	chr14: 100881886-100881978	UACGGUGAGCCUGUCAUUAUUCAUCAUGAUGGGCUCCUCGGUGU	
	*mmu*	Yes (miR-337, miR-3544, miR-665, miR-3070-1, miR3070-2, miR-431, miR-127, miR-434, miR-432, miR-3071, miR-136)	chr12: 109591715-109591838	AUCAUGAUGGGCUCCUCGGUGU	UACGGUGAGCCUGUCAUUAUUC
miR-487b	*hsa*	Yes (miR-376c, miR-376a-2, miR-654, miR-376b, miR-300, miR-1185-1, miR-1185-2, miR-381, miR-539, miR-889, miR-544a, miR-655, miR-487a, miR-382, miR-134, miR-668, miR-485, miR-323b)	chr14: 101046455-101046538	GUGGUUAUCCCUGUCCUGUUCGAAUCGUACAGGGUCAUCCACUU	
	*mmu*	Yes (miR-495, miR-667, miR-376c, miR-654, miR-376b, miR-376a, miR-300, miR-381, miR-539, miR-889, miR-544, miR-382, miR-134, miR-668, miR-485, miR-453)	chr12: 109727333-109727414	AAUCGUACAGGGUCAUCCACUU	UGGUUAUCCCUGUCCUCUUCG
miR-496	*hsa*	Yes (miR-487a, miR-382, miR-134, miR-668, miR-485, miR-323b, miR-154, miR-377, miR-541, miR-409, miR-412, miR-369, miR-410, miR-656)	chr14: 101060573-101060674	UGAGUAUUACAUGGCCAAUCUC	
	*mmu*	Yes (miR-544, miR-382, miR-134, miR-668, miR-485, miR-453, miR-154, miR-377, miR-541, miR-409, miR-412, miR-369, miR410, miR-3072)	chr12: 109739119-109739197	UGAGUAUUACAUGGCCAAUCUC	AGGUUGCCCAUGGUGUGUUCA
miR-99a	*hsa*	Yes (miR-let-7c)	chr21: 16539089-16539169	AACCCGUAGAUCCGAUCUUGUG	CAAGCUCGCUUCUAUGGGUCUG
	*mmu*	Yes (miR-let-7c)	chr16: 77598936-77599000	AACCCGUAGAUCCGAUCUUGUG	CAAGCUCGUUUCUAUGGGUCU

*hsa, human; mmu, mouse; chr, chromosome. Red highlights nucleotide differences between humans and mice*.

Of note, miR-17, miR-18a/b, miR-19a/b, miR-20b, miR-25, and miR106a are members of the three paralog clusters: miR-17/92, miR-106a/323, and miR-106b/25. These clusters contain miRNAs that are very comparable, regulate similar sets of genes, and have overlapping functions (24). Their genomic organization is highly conserved, which suggests important functions and coordinated regulations. Overall, 7 and 4 miRNAs in human and mouse, respectively, were overexpressed in classical monocytes (Figure 1B).

The miR-17/92 cluster is a well-described cluster that plays a role in immune responses (25). In the lymphocyte lineage, this cluster is expressed in B and T precursor cells, and its expression diminishes upon differentiation (26). In the monocytic lineage, monocyte hematopoiesis is affected by loss of the miR-17/92 cluster in humans but is unaffected in mouse (27). Human CD34^+^ hematopoietic progenitor cells differentiate *in vitro* into monocytes upon exposure to macrophage-colony stimulating factor; this differentiation leads to decreased expression of the miR-17/92 cluster, which is inversely correlated with upregulation of the transcription factor acute myeloid leukemia 1, a validated human target of miR-17. In addition, overexpression of the miR-17/92 cluster delays terminal differentiation of monocytes, and its inhibition accelerates differentiation (28). Differences between human and mouse data may be caused by species-specific differences and/or the fact that experiments were performed *in vitro* or *in vivo*. Using genetic mouse models, deletion of the miR-17/92 cluster and its paralog miR-106b/25 led to severe developmental defects, so these two miRNA clusters may act synergistically on cell survival to control embryonic development (27). Moreover, miR-17, miR-20a, and miR-106a, which belong to the two cluster paralogs miR-17/92 and miR-106a/363, regulate macrophage infiltration, phagocytosis, and proinflammatory cytokine secretion via targeting signal-regulatory protein alpha expression, both *in vitro* and *in vivo* (29).

Despite rare reports describing these 25 miRNAs in the context of monocyte subsets (see below), many more exist on their role in monocyte differentiation or inflammation processes. For example, some miRNAs are involved in macrophage polarization; one is miR-148a-3p, which promotes macrophage 1 (M1) polarization and inhibits M2 polarization upon Notch activation (30). Others are involved in osteoclastogenesis [e.g., the miR-29 family regulates osteoclast commitment and migration (31)]. MiR-223 is upregulated during granulopoiesis and fine-tunes the differentiation of myeloid precursors into granulocytes or monocytes and negatively controls the activity of NLRP3 inflammasome in these cell types (32). Also, miR-433 negatively regulates the hematopoietic cell proliferation by directly targeting interferon-induced guanylate-binding protein 2 (33), and miR-130a regulates the expression of macrophage pro-fibrogenic genes in chronic inflammation (34).

### Putative Function of miRNAs Specific to Human Monocyte Subsets

**Figure 2:**
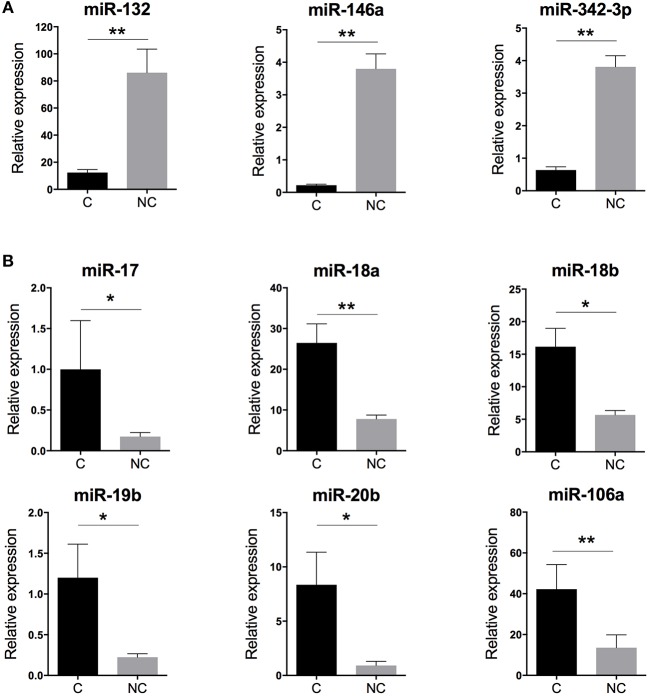
Validation of the human monocyte subset-specific miRNA-based signature. Blood samples from healthy donors (*n* = 7) were collected from the French Blood Establishment (EFS). After Ficoll-Paque density gradient, classical (C) CD14^++^CD16^−^ and non-classical (NC) CD14^+^CD16^++^ monocyte subsets were FACS sorted with >97% purity (Montpellier RIO Cytometry platform). Total RNA was extracted from both monocyte subsets by using a miRNeasy kit and the automatized QIAcube procedure (QIAGEN). MiRNA expression was quantified by using multiplexed TaqMan RT-qPCR (Life Technology). **(A)** Quantification of the three miRNAs overexpressed in non-classical vs. classical monocytes. **(B)** Quantification of the six miRNAs overexpressed in classical vs. non-classical monocytes. Data are mean ± SD and differences were compared by non-parametric Mann-Whitney test (^*^p < 0.05, ^**^p < 0.01).

With human miRNome data from classical and non-classical monocytes (Figure 1A), we quantified the expression of the nine miRNAs commonly deregulated between both subsets by using RT-qPCR and new samples. We confirmed the overexpression of miR-132, miR-146a, and miR-342-3p in human non-classical vs. classical monocytes and the overexpression of miR-17, miR-18a, miR-18b, miR-19b, miR-20b, and miR-106a in classical vs. non-classical monocytes (Figure 2).

**Figure 3:**
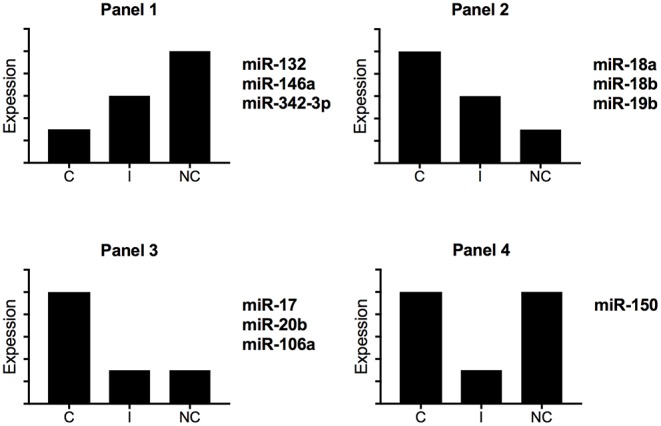
Schematic representation of miRNA expression profiles for human monocyte subsets. By using miRNome data from the study of Zawada et al. (22), we identified four different expression profiles. C, classical monocytes CD14^++^CD16^−^; I, intermediate monocytes CD14^++^CD16^+^; NC, non-classical monocytes CD14^+^CD16^++^.

Zawada et al. hypothesized that intermediate monocytes have a distinct miRNA profile as compared with classical and non-classical monocytes and identified 38 miRNAs differentially expressed in intermediate monocytes vs. both classical and non-classical monocytes (22). Figure 3 gives a schematic representation of the miRNA expression profile patterns for the three human monocyte subsets in the Zawada et al. study. Of note, miRNAs in panel 1 with gradually increasing expression from classical to intermediate and non-classical monocyte subsets included the three miRNAs that we found upregulated in our comparative study (miR-132, miR-146a, and miR-342-3p; Figure 2A). Panels 2 and 3, showing decreasing expression from classical to non-classical monocytes, displaying (panel 2) or not (panel 3) differences between intermediate and non-classical monocytes, contained the six miRNAs that we found downregulated in our comparative study (Figure 2B). Zawada et al. identified a fourth panel, including miR-150, with downregulated miRNAs in intermediate monocytes as compared with both classical and non-classical monocytes (*p* < 10^−10^, and > 10-fold difference in expression). Our Venn diagram analysis identified miR-150 as the only miRNA with differential expression between classical and non-classical monocyte datasets in mouse but not human datasets (Figure 1A), which agrees with the Zawada et al. miRNome data (Panel 4).

**Figure 4:**
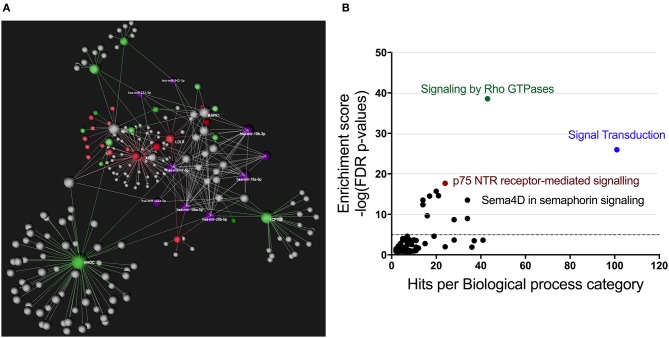
Gene ontology analysis of genes putatively targeted by monocyte subset-specific miRNAs. **(A)** Using OmicsNet, a force-directed sub-network was constructed for the nine miRNAs with differential expression between classical and non-classical human monocytes (color violet) and their putative target genes extracted from a list of 182 genes with differential expression in classical and non-classical monocyte subsets. Red and green represent genes up- and downregulated, respectively, in classical vs. non-classical monocytes. Genes in gray are those that link genes putatively targeted by miRNAs or are associated in the network. **(B)** By using Reactome pathway data, we plotted genes with differential expression between monocyte subsets as the number of genes for the respective biological function category (x-axis) against the enrichment score for log10 of *p*-value (y axis).

Using OmicsNet, a web-based tool for the creation and visual analysis of biological networks (35), we uploaded the list of nine monocyte subset-specific miRNAs identified in our human analysis together with the list of 182 genes found overexpressed in classical or non-classical monocytes identified in five independent transcriptomic microarray datasets (36). We aimed to create and merge different types of biological networks that could provide a clue to the pathways involved in the functional heterogeneity of monocyte subsets. Figure 4A shows the 3D OmicsNet biological networks highlighting connections between the nine miRNAs and putatively targeted genes according to the TarBase software [Table 2; (37)]. Gene Ontology analysis with the Reactome pathway database (38) showed enrichment of biological process categories such as signal transduction, small GTPases of the Rho family (Rho GTPases), p75 NTR receptor-mediated and Sema4D in semaphorin signaling (Figure 4B and Table 3). The trafficking of monocytes into tissues requires the activation of integrins via signal transduction induced by Rho GTPases such as RHOA or RAP1, which results in cell adhesion to the blood-vessel wall (39). Rho-GTPases are key regulators of cellular actomyosin dynamics and are therefore considered pharmacological targets for restricting leukocyte motility, including monocytes, in inflammatory disorders (40). A comparison of protein expression based on cell maturity (from pro-monocyte to monocyte and to macrophage lineages) suggested that Rho proteins are readily available for signaling events in response to numerous activating cues (41). Human CD100/Sema4D belongs to a large family of membrane-bound proteins named Semaphorins that are involved in numerous functions, including axon guidance, morphogenesis, carcinogenesis, and immunomodulation; Sema4D in particular influences monocyte migration (42). Resident microglia and infiltrated peripheral monocytes are two main types of immune cells in the central nervous system that control the inflammation process. Recently, the p75 neurotrophin receptor (p75NTR) was found to play a role in the peripheral expansion and central nervous system trafficking of pro-inflammatory monocytes (43).

**Table 2:** List of genes putatively targeted by the nine monocyte subset-specific miRNAs.

**miR-17**	**miR-18a**	**miR-18b**	**miR-19b**	**miR-20b**	**miR-106a**	**miR-132**	**miR-146a**	**miR-342-3p**
AIB1	AIB1		AIB1	AIB1	AIB1	AIB1	AIB1	AIB1
APP					APP			
CCND1	CCND1	CCND1		CCND1	CCND1			
CRK				CRK	CRK	CRK		
DCAF8	DCAF8	DCAF8	DCAF8	DCAF8	DCAF8			
F2RL1				F2RL1	F2RL1			
FAS	FAS		FAS		FAS		FAS	
GIGYF1	GIGYF1	GIGYF1	GIGYF1	GIGYF1	GIGYF1			
ITGA2	ITGA2	ITGA2	ITGA2	ITGA2	ITGA2			
								INSIG1
LDLR			LDLR	LDLR	LDLR	LDLR		
MAPK1			MAPK1	MAPK1	MAPK1	MAPK1		
MAPK14			MAPK14		MAPK14	NAP1L1		
NAP1L1								
PTEN	PTEN		PTEN	PTEN	PTEN			
							RAC1	
RHOC				RHOC	RHOC			
RLIM	RLIM	RLIM	RLIM	RLIM	RLIM			
RORA	RORA	RORA	RORA	RORA	RORA			
SMAD4	SMAD4		SMAD4	SMAD4	SMAD4		SMAD4	
TCF7L2				TCF7L2	TCF7L2			
TNRC6B	TNRC6B	TNRC6B	TNRC6B	TNRC6B	TNRC6B			
UBC	UBC			UBC				
WAC	WAC	WAC	WAC	WAC	WAC			WAC

**Table 3:** Gene ontology and functional pathway enrichment analysis.

**Pathway**	**Total**	**Expected false positives**	**Hits**	**–log (FDR *p-*values)**
Sema4D induced cell migration and growth-cone collapse	29	0.634	14	22.1
Sema4D in semaphorin signaling	34	0.743	14	18.8
NRAGE signals death through JNK	45	0.984	17	17.3
Rho GTPase cycle	123	2.69	43	16.0
Signaling by Rho GTPases	123	2.69	43	16.0
Cell death signaling via NRAGE NRIF and NADE	62	1.36	20	14.7
Lipoprotein metabolism	22	0.481	7	14.6
p75 NTR receptor-mediated signaling	85	1.86	24	12.9
G alpha (12/13) signaling events	80	1.75	21	12.0
Semaphorin interactions	72	1.57	16	10.2
Signaling by NGF	290	6.34	34	5.4
Axon guidance	292	6.38	28	4.4
Platelet activation signaling and aggregation	220	4.81	19	4.0
Developmental Biology	417	9.12	34	3.7
Signal Transduction	1,690	36.9	101	2.7

*The top 10 terms were selected according to false discovery rate (FDR)-adjusted p-values*.

Although these pathways and putative target genes have not yet been validated and functionally studied, further investigating their implication will increase our understanding of the functional heterogeneity of monocyte subsets.

## Role of miRNAs in Monocyte Subsets

Since the first description of blood monocytes in the early 2000s as a heterogeneous population of leukocytes displaying different phenotypic markers, homing properties, and immune functions (44), the scientific community has tried to dissect the role of individual subsets by identifying protein-encoding genes that specifically control the development and function of each sub-population. For example, the lineage-defining transcription factor nuclear receptor 4a1 (*Nr4a1*) was found essential for Ly6C^low^ monocyte development because *Nr4a1*^−/−^ mice lack Ly6C^low^ monocytes (45). Because *Nr4a1* regulates inflammatory gene expression and differentiation of Ly6C^low^ monocytes, the functions of Ly6C^high^ monocytes can be studied independently *in vivo* by using *Nr4a1*^−/−^ mice (46). The same expectations have been expressed for miRNA-encoding genes. However, few miRNAs have been identified (see previous section), and only three have been thoroughly studied *in vivo* by using genetic models. The first identified and most studied is miR-146a. In 2012, the group of Mikael Pittet showed that miR-146a is the highest differentially expressed miRNA between Ly6C^high^ and Ly6C^low^ monocytes (20). Also, until 2018, it remained the only miRNA described as regulating the functional heterogeneity of monocyte subsets.

### Control of Innate Immune Response to Infections

For many years, miR-146a has beenknownas anegativeregulator of inflammation inmyeloid cells (26, 47). In-depth characterization of miR-146a^−/−^ mice revealed decreased hematopoietic stem cell homeostasis during chronic inflammation, dysregulated hematopoietic stem cell differentiation toward myeloid cells, and abnormal myeloproliferation (48). The group of Pittet showed that miR-146a expression was inducible only in Ly6C^high^ monocytes upon inflammatory stimuli, reaching levels comparable to those in Ly6C^low^ monocytes in basal conditions. Lack of miR-146a in mice did not alter the development of monocyte subsets but markedly amplified the inflammatory response of Ly6C^high^ monocytes upon bacterial challenge by targeting RelB, a non-canonical NF-κB family member highly conserved between mice and humans. This amplification of the inflammatory response is not due to more pro-inflammatory cytokine production per cell but rather to an expansion of Ly6C^high^ monocytes in the bone marrow and their increased trafficking to inflamed tissue during acute bacterial challenge because of high expression of CCR2 and responsiveness to monocyte chemoattractant protein 1-mediated chemoattraction. This interesting result parallels the fact that neither TNF receptor associated factor six nor interleukin 1 receptor associated kinase 1 expression was modified by miR-146a in Ly6C^high^ monocytes (20, 49), but they were modified in monocytic cell lines (47). Overall, by maintaining a low level of miR-146a, Ly6C^high^ monocytes can rapidly proliferate into the bone marrow to be the first mobilized cells to egress into the circulation and rejoin the site of bacterial attack. In contrast, Ly6C^low^ monocytes remain insensitive to this type of environmental danger because of constitutive high expression of miR-146a. Thus, the gradual increase of miR-146a expression in Ly6C^high^ monocytes upon stimulation acts as a negative feedback loop that represses proliferation and prevents overwhelming amplification of the inflammation by so-called inflammatory Ly6C^high^ monocytes within the injured tissue, which would be deleterious. However, this study does not answer the question of the role of miR-146a in Ly6C^low^ monocytes.

RelB can directly bind with the aryl hydrocarbon receptor (AHR) that supports the xenobiotic-detoxifying pathway, the AHR nuclear translocator like 1 (also named Bmal1) partner of Clock that regulates the circadian rhythm, and the bioenergy sensor sirtuin 1 (Sirt1) to integrate acute inflammation with changes in metabolism and mitochondrial bioenergetics. Finally, RelB is involved in chromatin modifications, and low RelB expression recapitulates the formation of silent heterochromatin upon endotoxin tolerance conditions, further halting inflammatory signaling (50). Although these functions have not all been investigated in terms of monocyte heterogeneity and/or miRNA context, the miR-146/Relb axis might be the missing link with Bmal1-dependent regulation of Ly6C^high^ diurnal variations controlling their trafficking to sites of inflammation (51), AHR-dependent regulation of Ly6C^high^ monocyte-derived DC differentiation (52), and Sirt1-mediated inhibition of the pro-inflammatory macrophage activation (53). These are interesting areas to be addressed.

### Control of the Inflammatory Response in the Context of Atherosclerosis

In 2015, the group of Robert Raffai showed that apolipoprotein E (ApoE) expression was higher in Ly6C^low^ than Ly6C^high^ monocytes (54). The expression of ApoE in monocytes had an anti-inflammation effect by enhancing the purine-rich PU-box binding protein 1-dependent miR-146a transcription, thereby reducing Ly6C^high^ monocytosis, NF-κB–mediated inflammation, and atherosclerosis in the setting of hyperlipidemia. Thus, increasing miR-146a expression in Ly6C^high^ monocytes might have therapeutic application in atherosclerosis. Also, miR-146a may play a role in controlling the proliferation of Ly6C^high^ monocytes. This finding contradicts the general concept that monocytes are non-proliferating cells (55) but agrees with studies observing Ly6C^high^ monocytosis in bone marrow and blood, in different pathological contexts (20, 49, 54).

### Role in Bone Erosion and Formation

RelB also promotes the differentiation of myeloid precursors into DCs and OCs and activates the transcription of pro-inflammatory genes in response to immune signals and environmental stressors. Monocyte subset-specific differences in miR-146a expression, together with the well-described role of miR-146a as a negative feedback regulator of inflammation and osteoclastogenesis in myeloid cells (56, 57) and reduced level of miR-146a expression in Ly6C^high^ monocytes, might explain why Ly6C^high^ monocytes are prone to egress from the bone marrow upon inflammatory stimuli and differentiate into DCs and OCs upon entry into the inflamed site. Also, in contrast, this information might also explain why by maintaining constitutively high levels of miR-146a, Ly6C^low^ monocytes are prevented from differentiating into DCs and OCs. Indeed, monocyte subsets have a differential contribution to osteoclastogenesis (16). The capacity of the Ly6C^high^ subset but not Ly6C^low^ subset to develop into OCs has been recently attributed to low miR-146a expression (49). Indeed, our group showed that classical monocytes display reduced miR-146a expression in both arthritic humans and mice as compared with healthy individuals; *in vivo* delivery of miR-146a mimics into Ly6C^high^ monocytes using a specific delivery system that spares Ly6C^low^ monocytes (58) rescued RelB expression in Ly6C^high^ monocytes, reduced their capacity to differentiate into OCs and reduced inflammatory-mediated bone erosion in an experimental model of arthritis. This is the first work to provide an *in vivo* proof of concept for a therapeutic strategy design targeting a subset-specific miRNA. Whether miR-146a plays other roles in Ly6C^high^ monocytes and investigating its Ly6C^low^-specific function(s) remains for further investigation. Our transcriptomic analyses comparing both monocyte subsets sorted from miR-146a^+/+^ and miR-146^−/−^ mice showed that miR-146a modulates the expression of 1,000 genes in Ly6C^high^ monocytes but only 100 genes in Ly6C^low^ monocytes (49), which suggests that beyond osteoclastogenesis, miR-146a may play other roles in Ly6C^high^ functions but not many in Ly6C^low^ monocytes.

### miRNA Function in Monocyte Subset Differentiation

Recently, the group of Stéphane Potteaux identified an miRNA critical for generating Ly6C^low^ monocytes. The authors observed increased expression of miR-21 in non-classical Ly6C^low^ monocytes of atherosclerotic ApoE^−/−^ mice, which mediated their higher number and lifespan in this model (59). The frequency of Ly6C^low^ monocytes in blood, bone marrow and spleen was markedly reduced in ApoE^−/−^ miR-21–deficient mice. Consequently, Ly6C^low^ monocyte numbers were reduced in the atherosclerotic aorta because of increased susceptibility to apoptosis. However, miR-21 deficiency did not affect trafficking of Ly6C^high^ monocytes nor their number in atherosclerotic aortas or the size of lesions but was associated with the presence of more pro-inflammatory macrophages in plaque, increased necrotic core, deficient efferocytosis, and increased macrophage death. This work reveals a role for miR-21 in atherosclerosis development and reveals the proof of concept that inhibiting miR-21 in monocytes might have relevant therapeutic application in atherosclerosis. However, many questions remain, including the target gene(s) that mediates the observed phenotype in ApoE^−/−^ mice, whether miR-21 plays a role in Ly6C^low^ monocytes under non- ApoE^−/−^ conditions, and which role it plays (if any) in the biology of Ly6C^high^ monocytes. In addition, because miR-21 controls macrophage polarization, apoptosis, and efferocytosis (60), determining which of these functions is affected in the context of monocyte subsets would be of interest.

In 2018, the group of Eric Solary identified miR-150 as overexpressed in Ly6C^low^ monocytes as compared with Ly6C^high^ monocytes and critical for promoting the terminal differentiation of classical monocytes into non-classical monocytes in both humans and mice (61). The authors found a defect of Ly6C^low^ monocytes in miR-150–deficient mice that did not affect the total number of monocytes in peripheral blood and bone marrow and was due to the un-repressed expression of Tet methylcytosine dioxygenase 3 (*Tet3*) gene. Tet3 is a dioxygenase that binds DNA and mediates demethylation but also promotes open chromatin independent of its catalytic function. Overall, Tet3 enhances transcription and gene expression, especially during changes in cellular identity (62). Thus, high *Tet3* expression in Ly6C^high^ monocytes due to low expression of miR-150 in this subset prevented their differentiation into Ly6C^low^ monocytes. This finding has important clinical implications because reduced expression of miR-150 was also found in peripheral-blood CD14^+^ monocytes, mostly classical monocytes, of patients with chronic myelomonocytic leukemia. Thus, monitoring the repartition of monocyte subsets is now an international recommendation as a diagnostic tool for patients with monocytosis to distinguish between chronic myelomonocytic leukemia and reactive monocytosis (63).

## Conclusion

In this review, we give an overview of the state-of-the-art research of miRNAs that are differentially expressed between monocyte subsets and how they affect monocyte functional heterogeneity, with descriptions of functional and *in silico* studies of specific miRNAs. Three miRNAs miR-146a, miR-21, and miR-150 with differential expression in classical vs. non-classical monocyte subsets were all first identified as immune system regulators. The three miRNAs are inducible mediators of anti-inflammatory responses in the myeloid lineage acting via negative feedback loops and leading to the resolution of inflammation. Thus, they represent switches from pro- to anti-inflammatory responses of real therapeutic potential.

**Figure 5:**
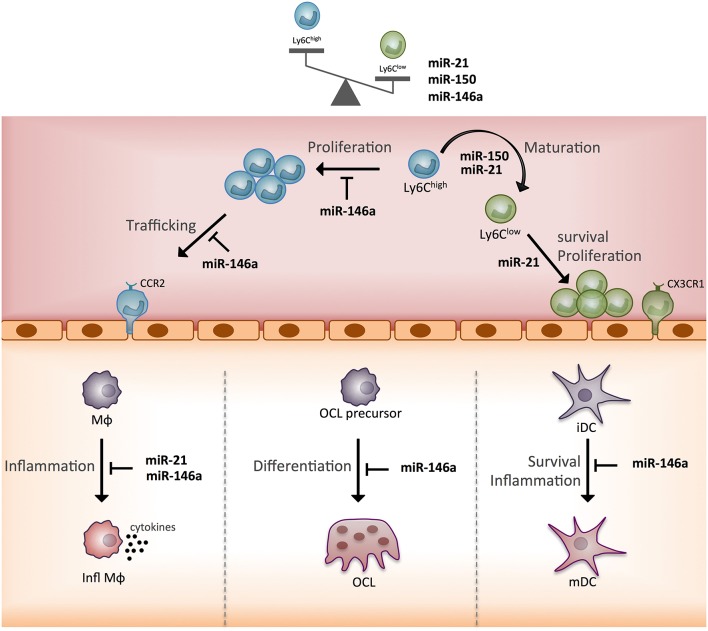
Schematic representation of the function of three monocyte subset-specific miRNAs. With the three miRNAs showing differential expression in monocyte subsets that were functionally studied in mouse models, we propose a scheme outlining their role. MΦ, macrophage; Infl MΦ, inflammatory macrophage; OC, osteoclast; iDC, immature dendritic cell; mDC, mature dendritic cell; CCR2, C-C chemokine receptor type 2; CX3CR1, CX3C chemokine receptor 1.

In terms of the functional heterogeneity of monocyte subsets, the few studies described bring new valuable insights into the role of the three miRNAs. Indeed, by identifying new target genes and functions that discriminate Ly6C^low^ and Ly6C^high^ mouse monocytes, these studies helped specify miRNA-based mechanisms for the commitment of monocytes to a cellular subset fate and related functional specificity (Figure 5). Of note, miR-146a, miR-21, miR-150 all show greater expression in Ly6C^low^ rather than Ly6C^high^ monocytes, and their expression is increased by inflammation. The reported role for miR-146a concerns mainly Ly6C^high^ monocyte inflammatory functions, and that of miR-21 and miR-150 concerns the generation of Ly6C^low^ monocytes. However, so far, all studies have been performed with Ly6C^high^ monocytes, thus future studies need to work with Ly6C^low^ monocytes to address the role of miRNAs in the function specific to Ly6C^low^ monocytes. In addition, the role of miRNAs in the commitment of monocyte subsets to OCs remains poorly explored and deserves further investigations (64).

Finally, we must revisit this knowledge in light of recent works using single-cell RNA sequencing of human blood monocytes (65), high-dimensional mass cytometry (66), and ontogeny study (67), under steady state or pathological conditions (68), which broadens our perspectives by identifying new monocyte subsets and further underlines the control of monocyte plasticity by miRNAs and their target genes. Recently, 29 human immune cell types have been characterized by RNA sequencing and flow cytometry, and mRNA heterogeneity and abundance appeared to be cell type-specific (69). Most miRNAs act as rheostats, refining the expression of hundreds of genes to enhance cell differentiation. Thus, miRNA detection in single-cell monocytes is needed to understand the biology of the heterogeneity of monocytes and to propose new strategies for disease treatment and diagnosis.

## Data Availability

The datasets analyzed for this study can be found in the NCBI gene expression omnibus, GSE137729 and GSE137730 for mouse and human data sets, respectively.

## Ethics Statement

The studies involving human participants were reviewed and approved by the local human ethics committee (ID RCB 2008-A01087-48) and with the code of ethics of the world medical association. Written informed consent for participation was not required for this study in accordance with the national legislation and the institutional requirements.

## Author Contributions

ID-R contributed to the design of the work, analysis and interpretation of the data, wrote the review, and designed the figures and tables. MR and CP contributed to the acquisition and analysis of the data and revised the manuscript. FA contributed to the conception of the work and interpretation of the data, wrote the review, and designed the figures. All authors gave approval for publication of the content.

### Conflict of Interest Statement

The authors declare that the research was conducted in the absence of any commercial or financial relationships that could be construed as a potential conflict of interest.
